# The COVID-19 pandemic and cannabis use in Canada―a scoping review

**DOI:** 10.1186/s42238-023-00196-7

**Published:** 2023-07-31

**Authors:** Kelda Newport, Lisa Bishop, Jennifer Donnan, Shefali Pal, Maisam Najafizada

**Affiliations:** 1 School of Pharmacy, Memorial University of Newfoundland and Labrador, St. John’s, NL A1B 3V6 Canada; 2 Faculty of Medicine, Memorial University of Newfoundland and Labrador, 300 Prince Phillip Drive, NL A1B 3V6 St. John’s, Canada

**Keywords:** COVID-19, Cannabis, Scoping review, Canada

## Abstract

**Background:**

Since the start of the COVID-19 pandemic in Canada, the cannabis industry has adapted to public health emergency orders which had direct and indirect consequences on cannabis consumption. The objective of this scoping review was to describe the patterns of consumption and cannabis-related health and safety considerations during the COVID-19 pandemic in Canada.

**Methods:**

For this scoping review, we searched four electronic databases supplemented with grey literature. Peer-reviewed or pre-print studies using any study design and grey literature reporting real-world data were included if published in English between March 2020 and September 2021 and focused on cannabis and COVID-19 in Canada. A content analysis was performed.

**Results:**

Twenty-one studies met the inclusion/exclusion criteria. Study designs included cross-sectional surveys (*n* = 17), ecological study (*n* = 1), conceptual paper (*n* = 1), longitudinal study (*n* = 1), and prospective cohort study (*n* = 1). Most were conducted solely in Canada (*n* = 18), and the remaining included global data. Our content analysis suggested that cannabis consumption during the pandemic varied by reasons for use, consumers’ age, gender, and method of consumption. Health and safety impacts due to the COVID-19 pandemics included increased mental illness, increased emergency visits, and psychosocial impacts.

**Discussion:**

This scoping review suggested that the impact of the pandemic on cannabis consumption in Canada is more complex than simplistic assumptions of an increase or decrease in consumption and continues to be difficult to measure. This study has explored some of those complexities in relation to reasons for use, age, gender, method of consumption, and health impacts. This scoping review is limited by focusing on the breadth compared to depth.

**Conclusions:**

Legalizing nonmedical use of cannabis in Canada in 2018 has had its challenges of implementation, one of which has been the changing context of the society. The findings of this study can help inform cannabis policy updates in Canada as the country is reaching its fifth year of legalizing nonmedical use of cannabis.

## Introduction

Since the legalization of nonmedical use of cannabis in Canada in 2018, the cannabis industry has evolved rapidly (Rotermann 2020). The COVID-19 pandemic and the federal and provincial public health emergency orders implemented in response to the pandemic affected the cannabis consumption patterns in a multitude of ways. During the pandemic, provinces and territories have ordered full lockdowns and other restrictive measures on businesses to help curtail the spread of the virus (Canadian 2021). Businesses have also had to adapt to the changing environment. Physical retail outlets took steps to reduce in-person interactions and close contact primarily by implementing physical distancing measures within stores, increasing sanitation measures, reducing operating hours, and where possible providing curbside pickup or online sales (Canadian 2022).

The COVID-19 pandemic and associated public health measures implemented to reduce transmission of the SARS-CoV‐2 virus has had direct and indirect consequences on cannabis consumption, many of which are preliminary and continue to be researched (Statistics 2021; Canadian 2020; Canadian 2021). In Canada, the sale of cannabis falls under provincial and territorial jurisdiction and during the initial pandemic lockdowns, most jurisdictions either declared or treated the non-medical retail sale of cannabis as an essential service (Canadian 2021). Retail sales of cannabis, for example, increased across Canada except for Prince Edward Island after the initial waves of the pandemic (Myran 2020). One poll conducted earlier in the pandemic showed that cannabis consumption in Canada remained stable (Canadian 2020), while a second poll indicated that the frequency and quantity of cannabis use increased by over 30% among those who consumed cannabis (Canadian 2020). A Canadian Centre on Substance Use and Addiction (CCSA) study in 2022 reported that purchases from licensed retailers had been increasing as the two most common avenues of purchase in 2020 were from legal physical and online stores (Canadian 2022). Moreover, the consumption method has been shifting slightly from smoking to edible products as they become more available through legal channels (Canadian 2022).

The impact of the pandemic on cannabis consumption in Canada and, specifically, whether health- and socioeconomic-related changes and stresses have impacted patterns of use, is of particular interest as we enter the fifth year of cannabis legalization in Canada―a time and opportunity for policy adjustment. The objective of this scoping review was to describe the patterns of consumption and cannabis-related health and safety considerations during the COVID-19 pandemic in Canada. A scoping review was conducted to systematically map the research in this area, identify gaps in existing knowledge, and make recommendations for future research.

## Methods

Due to the evolving nature of the pandemic with rapidly emerging evidence, a scoping review was deemed to be the most appropriate method (Levac et al. 2010). We adopted the five-stage scoping review methodology developed by Arksey and O’Malley: (1) identify the research question; (2) identify the relevant studies; (3) indicate the inclusion and exclusion criteria; (4) chart the data; and (5) collate, summarize, and report the results. Arksey and O’Malley define a scoping review as “a technique to ‘map’ relevant literature in the field of interest … [which] tends to address broader topics where many different study designs might be applicable …” (Arksey and O’Malley 2005). The Joanna Briggs Institute (JBI) guidelines on scoping reviews were also adopted to guide our methodology and align with current reporting standards in evidence synthesis (Peters et al. 2015). Reporting was also informed by the PRISMA Extension for Scoping Reviews (Tricco et al. 2018).

This review was initiated with the question “What impact has the COVID-19 pandemic had on cannabis consumption and health and safety issues related to cannabis in Canada?” The search strategies were developed in consultation with a librarian and through team discussion. Electronic databases (PubMed, CINAHL Plus, Embase, and Web of Science) were searched according to the search strategy presented in Table 1 and supplemented with searching Google Scholar, Public Health Agency of Canada website, and Google search engine for grey literature.

**Table 1:** Search strategy

PubMed, EMBASE, Web of Science, and CINAHL Plus were searched using free texted terms:	
Cannabis OR Weed OR Pot OR Marijuana OR Tetrahydrocannabinol OR Cannabidiol OR Cannabinoid	
AND	
COVID-19 OR Pandemic OR Coronavirus OR SARS COV-2 OR 2019-NCOV OR COV-19	
AND	
Canada OR Canadians OR Provinces (searched by individual province name)	

Article titles and abstracts resulting from the database and grey literature searches were screened independently by two authors (KN, SP). Full texts were then screened by both authors for potentially relevant publications. Disagreements on study selection and data extraction were resolved by consensus and discussion with a third author (MN) when necessary. The original search was conducted in May 2021 and updated on September 24, 2021.

Peer-reviewed or pre-print studies of Canadian data using any study design and grey literature reporting Canadian real-world data were included if they were published in English between March 1, 2020, and September 24, 2021, and focused on cannabis and COVID-19 in Canada. We broadly searched for literature related to cannabis, and although we did not intentionally search for medical cannabis, we included papers that reported on medical and non-medical cannabis. Media reports, editorials, opinion pieces, blog posts, and pharmacological papers were excluded. Papers in which specific cannabis data could not be separated from the aggregate data were also excluded. Inclusion and exclusion criteria are listed in Table 2.

**Table 2:** Inclusion and exclusion criteria

Inclusion criteria	Exclusion criteria
Data focusing on cannabis and COVID-19 in Canada: ● Peer-reviewed or pre-print studies of Canadian data using any study design OR ● Grey literature reporting Canadian real world Published in the English Language Published between March 1, 2020, and September 24, 2021	Specific cannabis data could not be separated from the aggregate data Media reports, editorials, opinion pieces, and blog posts Pharmacological papers Full article not accessible

A comprehensive data charting form was developed to extract relevant information from included sources of evidence. Abstracted data on study characteristics included: study author(s), date of publication, publication journal, location(s) of study, study population, sample size, study type, objectives, and key findings. One author (KN) charted the data and all authors discussed the results and continuously updated the data-charting form in an iterative process.

A content analysis is a research method that can be used to identify patterns across qualitative data, which often provides frequency counts and allows for quantitative analyses of qualitative data (Braun and Clarke 2006). We conducted a thematic content analysis to identify, analyze, and report patterns and themes and to establish how the identified themes fit together to tell a narrative about the data (Braun and Clarke 2014). A paragraph was the unit of analysis in the coding process. A single paragraph was coded multiple times when needed. The codes were then merged into sub-themes and themes. An initial list of themes was provided to the authors but the authors could identify new themes from the data as well―a mix of a deductive and inductive approach.

## Results

**Fig. 1:**
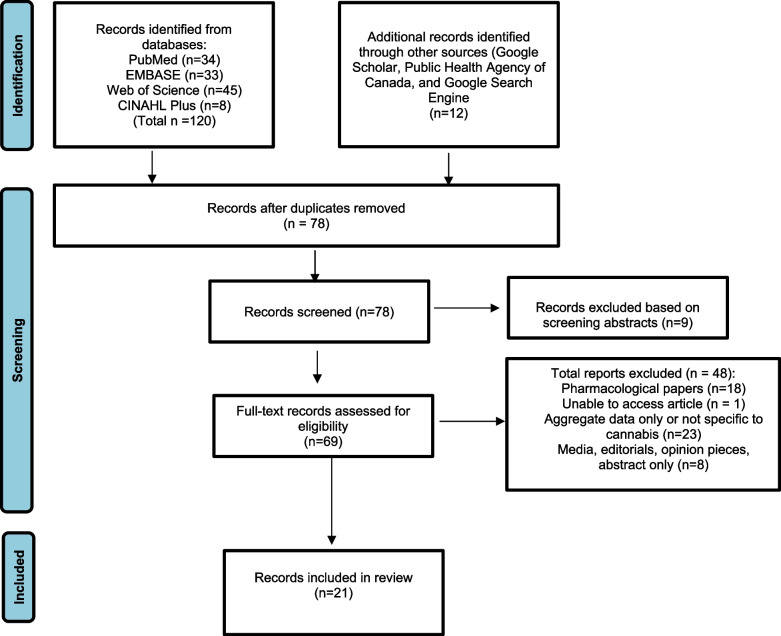
Flowchart of scoping review

A total of 132 references were initially identified, of which 69 full-text records were assessed for eligibility, and 21 records met the inclusion/exclusion criteria and were included in the scoping review (Fig. 1).

Seventeen of the included studies present results from cross-sectional surveys (Myran 2020; Canadian 2020; Currie 2021; Dozois 2021; Dumas et al. 2020; Farhoudian et al. 2020; Imtiaz et al. 2021; Joyce et al. 2020; Prowse et al. 2021; Rotterman 2020; Rotterman 2021; Statistics 2021; Turna 2021; Turna et al. 2021; Turner 2019; Vedelago et al. 2022; Zajacova et al. 2020). The remaining four include an ecological study (Canadian 2021), a conceptual model (Enns et al. 2020), a prospective cohort study (Leatherdale et al. 2021), and a longitudinal study (Bartel et al. 2020). Eighteen studies were conducted solely in Canada (Canadian 2021; Myran 2020; Canadian 2020; Currie 2021; Dozois 2021; Dumas et al. 2020; Imtiaz et al. 2021; Joyce et al. 2020; Prowse et al. 2021; Rotterman 2020; Rotterman 2021; Statistics 2021; Turner 2019; Vedelago et al. 2022; Zajacova et al. 2020; Enns et al. 2020; Leatherdale et al. 2021; Bartel et al. 2020), and the remaining three studies included global data, including Canadian data (Farhoudian et al. 2020), data from Canada and the United States (US) (Turna et al. 2021), or data collected from Canada, US, Brazil, and Italy (Turna 2021). A summary of the scoping review study data can be found in Table3 and characteristics of included studies can be found in Table 4.

**Table 3:** Summary of scoping review study data

Geographic locations of studies	Number of studies	%
Canada-wide	15	70
Ontario, Canada	1	5
Alberta, Canada	1	5
Ontario and Quebec, Canada	1	5
Canada and the USA	1	5
Canada, the USA, Brazil, and Italy	1	5
Worldwide (including Canada)	1	5
Study types	Number of studies	%
Cross-sectional surveys	17	80
Ecological study	1	5
Conceptual model	1	5
Longitudinal study	1	5
Prospective cohort	1	5

**Table 4:** Characteristics of included studies

Author(s) and date	Study design	Location of study	Population sample	Timeframe	Objective(s)
(Bartel et al. 2020)	Longitudinal study―online survey	Canada	70 individuals between 19 and 25 years of age	March 23 and June 15, 2020	Investigate the relationship between self-isolation due to COVID-19, using cannabis to cope with depression and cannabis consumption
(Canadian 2020)	Cross-sectional survey	Canada	A total of *n* = 2502 (T1) and *n* = 1507 (T2) online surveys were conducted, age 16 years and older	Time 1 (T1): October 13 to November 2, 2020 Time 2 (T2): November 19 to December 2, 2020	Monitor the ongoing impacts of the COVID-19 pandemic on mental health and substance use
(Canadian 2021)	Ecological study	Canada	Canadian residents age 10 and older	March to September 2020	Examine harm caused by substance use during the pandemic, analyzing emergency department visits and inpatient hospitalizations
(Currie 2021)	Cross-sectional	Alberta, Canada	933 community-based adults without a previous diagnosis of post-traumatic stress disorder (PTSD)	June 2020	Examine associations between pandemic-related PTSD symptoms and substance use among adults
(Dozois 2021)	Cross-sectional, MHRC’s national survey	Canada	1803 randomly selected adults (ages 18+)	April 22–28, 2020	Assess the relationship of the COVID-19 pandemic and levels of anxiety and depression.
(Dumas et al. 2020)	Retrospective cross-sectional online survey	Canada	1054 Canadian adolescents (aged 14–18)	April 4 - April 13, 2020	Assess how adolescent substance use has changed since the COVID-19 pandemic, in addition to key contexts and correlates of substance use during social distancing
(Enns et al. 2020)	Conceptual model	Canada	N/A	March and May 2020	Present a conceptual model of the possible effects of the COVID-19 pandemic on substance use and related harms
(Farhoudian et al. 2020)	Cross-sectional survey	Global (including Canada)	185 responses from 77 countries	April 5–May 10, 2020	Assess addiction medicine professionals’ perceived changes in drug and alcohol supply, price, use pattern and related complications during the COVID-19 pandemic
(Imtiaz et al. 2021)	Repeated cross-sectional	Canada	3012 adults in Canada	May 8–June 23, 2020	Characterize trends in cannabis use in the overall population and (2) characterize patterns of and identify risk characteristics associated with an increase in cannabis use among those who used cannabis
(Joyce et al. 2020)	Cross-sectional	Canada	508 mothers with children 0–8 years old	April 14–28, 2020	Examine substance use among mothers during the COVID-19 pandemic
(Leatherdale et al. 2021)	Prospective cohort	Quebec and Ontario, Canada	7496 students (grades 9 to 12 in Ontario and Secondary I–V in Quebec) participated in the 2020 online data collection in the 43 schools	May to July 2020	Examine the effect of the early stages of the COVID-19 pandemic period on youth cannabis use in the context of a natural experiment
(Myran 2020)	Cross-sectional time-series analysis of longitudinal sales data	Canada	Monthly per capita alcohol and cannabis retail sales for individuals ages 15 + in Canada	March–May 2020	Examine changes in per capita alcohol, cannabis, and other essential retail sales across Canada during the early phase of COVID-19 and associations between these changes and different jurisdictional approaches to drug control
(Prowse et al. 2021)	Cross-sectional survey	Ontario, Canada	366 undergraduate students from Carleton University	May–August 2020	Examine the impact of the COVID-19 pandemic on academics, social isolation, and mental health, as well as the extent to a variety of coping strategies have been implemented by study participants
(Rotterman 2020)	Survey by Statistics Canada CPSS Series 1; CCHS; NCS	Canada	CPSS : > 4600 people, aged 15 or older in the 10 provinces; CCHS: ~65,000 respondents aged 12 and older in 10 provinces and 3 territories (only those aged 15 and older and living in the 10 provinces were included); NCS: unknown	CPSS―March 23-April 3 2020; CCHS―2018; NCS―2019 fourth quarter	To report on how Canadians are coping with the COVID-19 pandemic and to estimate the rate of cannabis consumption in the past 3 months by self-perceived mental health
(Rotterman 2021)	Cross-sectional NCS	Canada	Canadians aged 15 and older in the 10 provinces. Provincial samples contained an average of 5540 respondents	The first quarter of 2018 and 2019 NCS, the fourth quarter of 2020	The primary objective of this study was to update the information to reflect changes in self-reported cannabis consumption and related behaviors, as well as examine how methods of consumption and products have been changing between 2018 and 2020
(Statistics 2021)	Cross-sectional: CPSS 6	Canadians living in the ten provinces	16,467 respondents aged 15 and older	January 25 to 31, 2021	To understand the possible impact of the COVID-19 pandemic on the use of substances, including alcohol, cannabis, opioids, and non-prescription substances
(Turna 2021)	Cross-sectional survey	Canada, the USA, Brazil, and Italy	1315 individuals	April 08–June 26, 2020	Compare the mental health burden on healthcare practitioners in different countries during the pandemic
(Turna et al. 2021)	Cross-sectional survey	Canada and the USA	632 individuals	April 8–June 11, 2020	Examine relevant risk factors for pandemic-related mental health issues
(Turner 2019)	Cross-sectional survey	Canada	809 adolescents, aged 12–18 years	June 17–July 31, 2020.	Explore the demographic and geographic distributions of suicidal ideation and deliberate self-harm and the associations of mental health and substance use with suicidal ideation and deliberate self-harm
(Vedelago et al. 2022)	Cross-sectional survey	Canada	137 Adults	April 30–May 4, 2020	Investigate internal cannabis use motives as a potential mediating factor between cannabis demand pre-declaration of COVID-19 emergency measures and cannabis use patterns and problems after the implementation of COVID-19 emergency measures
(Zajacova et al. 2020)	Cross-sectional survey	Canada	4383 adults age 25 and older	March 29 and April 3, 2020	Assess changes in health behaviors during the early stages of the pandemic and examine socio-demographic disparities associated with these changes

*CCSA* Canadian Centre on Substance Use and Addiction, *CCHS* Canadian Community Health Survey, *CIHI* Canadian Institute for Health Information, *CPSS* Canadian Perspectives Survey Series, *MHCC* Mental Health Commission of Canada, *MHRC* Mental Health Research Canada, *NCS* National Cannabis Survey, *N/A* Not applicable

### Content analysis

There was a mixture of information about the studies and factors relating to cannabis consumption during the pandemic. The population sample in the studies included in this review were general population adults [11], mothers [1], adolescents and youth (e.g., grade students, undergrad students) [5], healthcare practitioners [2], emergency department patients [1], and cannabis users [1]. One study found that cannabis use in the overall population remained stable during the early months of May and June 2020; however, they noted that during the same period, cannabis consumption among those who were prior cannabis consumers increased by half compared to before the pandemic (Imtiaz et al. 2021). Data from January 2021 from the Canadian Perspectives Survey Series (CPSS) 6: Impacts of COVID-19, which is a representative sample of the Canadian Population aged 15 years and older, also indicated that of those who had previously consumed cannabis, more than one third (34%) reported their consumption had increased during the pandemic (Statistics 2021).

Content analysis suggested that cannabis consumption during the pandemic varied by reasons for use, consumers’ age, gender, and method of consumption. The accentuated health impacts of cannabis consumption during the pandemic was also recorded. Our findings are described in more detail in the following sections and Tables 5, 6, 7, 8, 9 and 10.

**Table 5:** Main reasons for increased and decreased cannabis consumption during the pandemic

Reasons for increased consumption	Reasons for decreased consumption
Self-isolation, loneliness, and boredom	Lack of socializing opportunity
Anxiety, stress, and depression	Family responsibilities
Poor mental health	Higher education
Ease of access	Using with parents
Lack of schedule and availability of free time	Lack of access
Convenience	Negative effects of cannabis

**Table 6:** Key findings for reasons for increased and decreased cannabis use during the pandemic

Consumption pattern	Study	Key findings
Reasons for increased use	(Statistics 2021)	¬ CPSS 6 respondents reported stress (65%), boredom (58%), loneliness (39%), convenience (lack of regular schedule and being at home) (38%), and ease of access (29%) as the most frequently reported factors contributing to increased consumption of cannabis during the pandemic.
	(Bartel et al. 2020)	¬ Self-isolation and coping with depression were reported as motives for cannabis use during the pandemic. ¬ Cannabis consumption increased by 31% during the pandemic among those who always used cannabis to cope with depression. ¬ Participants who participated in self-isolation were using 20% more cannabis than those who did not self-isolate.
	(Imtiaz et al. 2021)	¬ Risk characteristics associated with higher odds of an increase in cannabis use included (odds ratio, 95% confidence intervals): • Residing in the central region of Canada (1.93, 1.03–3.62) • 18 to 29 years old (2.61, 1.32–5.17) or 30 to 49 years old (1.85, 1.07–3.19) • Less than a college or university education (1.86, 1.13–3.06) • Concern about the pandemic’s impact on personal finances (1.73, 1.00–3.00)
Reasons for decreased use	(Statistics 2021)	¬ Data from the CPSS 6 reported the following as reasons associated with decreased cannabis consumption during the pandemic: • The negative effects of cannabis (64%), • Decreased opportunities for socializing (28%), • Family and work obligations and responsibilities (16%)

**Table 7:** Age variation and cannabis use during the pandemic

Consumption pattern	Study	Key findings
Age	(Dumas et al. 2020)	¬ The percentage of cannabis using adolescents decreased for girls only (3% decrease, from 16.4 to 13.4%, *p* < .01); however, the frequency of cannabis use (average number of cannabis using days) increased significantly from pre-COVID to post-COVID (*F* (1, 1029) = 8.04, *p* = .01).
	(Imtiaz et al. 2021)	¬ Cannabis use in the overall population remained stable during the early months of May and June 2020; however, they found that during the same time cannabis use among those who used cannabis increased by half (55.65% at wave 1, 51.94% at wave 2, and 47.58% at wave 3) compared to before the pandemic (*p* > .05 for both chi-square and Cochran-Armitage tests).
	(Leatherdale et al. 2021)	¬ 27.3% (*n* = 241) of adolescents who reported past-year cannabis use reported that their use has increased because of COVID-19 ¬ 23.2% (*n* = 205) reported their cannabis use had decreased because of COVID-19. ¬ 27.4% (*n* = 242) of respondents reporting past-year cannabis use, reported using cannabis to cope with changes related to COVID-19.
	(Statistics 2021)	¬ Among respondents who had previously consumed cannabis, 43% of those aged 15 to 29, 20% of respondents aged 50 to 64, and 22% of those aged 65 or older reported increasing cannabis consumption during the pandemic

**Table 8:** Gender differences and cannabis use during the pandemic

Consumption pattern	Study	Key findings
Gender	(Leatherdale et al. 2021)	¬ Female youth appeared to either maintain or escalate use relative to males across all cannabis use outcomes modeled. ¬ The reduction in the expected escalation of cannabis use among males (− 1.6 (− 2.9, − 0.3) *p* = .016) relative to females (0.8 (− 0.2, 1.7) *p* = .138) during the initial COVID-19 period, which suggests cannabis use among males may be more socially driven than among females.
	(Dumas et al. 2020)	¬ The percentage of cannabis using adolescents decreased for girls only (3% decrease, from 16.4 to 13.4%, *p* < .01), yet the frequency of cannabis use increased significantly from pre-COVID to post-COVID (*F* (1, 1029) = 8.04, *p* = .01). Analysis revealed this increase was only significant for girls (*F* (1,799) = 8.04, *p* = .01) and not for boys (*F* (1, 225) ¼0.02, *p*¼0.88. More girls used substances with their parents (47%) and engaged with acquaintances through social posts (40%) in contrast to boys (30% and 24%, respectively).
	(Prowse et al. 2021)	¬ Coping with COVID-19 by using cannabis was associated with more negative impacts on schoolwork for males compared to females (*p* < .01). ¬ Cannabis use was associated with negative impacts on mental health for both males (*p*’s < 0.01) and females (*p*’s < 0.05), but the effects were stronger for males.

**Table 9:** Method of consumption and cannabis use during the pandemic

Consumption pattern	Study	Key findings
Method of consumption	(Bartel et al. 2020)	¬ Smoking was reported as the most commonly used method of consumption during the pandemic with over 75% of the sample reported using cannabis by smoking (59%) or vaping (17.1%).

**Table 10:** Health and safety impacts and cannabis use during the pandemic

Consumption pattern	Study	Key findings
Health and safety impacts	CIHI (2021)	¬ A substantial increase was observed in emergency departments visits and hospitalizations for cannabis-related harms from March to September 2020 compared with the same period in 2019: • 8% increase in emergency departments visits • 5% increase in hospitalizations
	Currie (2021)	¬ Pandemic-related PTSD symptoms were associated with more than a twofold increase in the odds of increased alcohol and/or cannabis use in the past month among both women [(2.58 (1.43, 4.63)] and men [2.73 (1.41–5.30)].

#### Reasons for cannabis consumption

Nine of the included studies reported either an increase or a decrease in the consumption of cannabis during the pandemic due to a variety of reasons, as summarized in Table 5. Some of the key findings with regard to reasons for increased and decreased cannabis consumption can be found in Table 6.

Multiple studies indicated that self-isolation, loneliness, and boredom (Statistics 2021; Enns et al. 2020), anxiety, stress, and depression (Canadian 2020; Statistics 2021; Bartel et al. 2020), and ease of access (Joyce et al. 2020; Enns et al. 2020)were all reasons for increased consumption. A 2021 survey of Canadians reported that stress (65%), boredom (58%), and loneliness (39%) during the pandemic were factors that contributed to increased consumption (Enns et al. 2020). Similar factors reported by the Canadian Center for Substance Abuse and Addiction (CCSA) included lack of schedule (57%), stress (56%), boredom (55%), and loneliness (16%) (Statistics 2021). Adults who were self-isolating had increased consumption by 20%, in contrast to those not self-isolating (Canadian 2020). Dozois (2021) found that with social distancing restrictions, 29% of individuals reported an increase in cannabis use (Dozois 2021). Similar to what was reported by adults, adolescents reported boredom and availability of free time as reasons for the increased use of cannabis during the pandemic (Imtiaz et al. 2021).

Canadians with self-assessed fair to poor mental health were two times more likely to have increased their cannabis consumption (Dozois 2021). Consumption also increased by 31% during the pandemic among those who always consumed cannabis to cope with depression (Canadian 2020). Mothers reported increased substance use to cope with anxiety, depression, or with younger children during the pandemic (Bartel et al. 2020).

The CPSS reported other factors related to access that contributed to increased consumption, which included convenience (38%) (e.g., lack of a regular schedule, at home often) and ease of access (29%) (e.g., availability of online shops, increase in retail stores, delivery, curbside pickup) (Statistics 2021).

Among respondents who reported decreased consumption over the pandemic in the Canadian national survey, the reasons included the negative effects of cannabis (64%), decreased opportunities for socializing (28%), and personal (e.g., family and work obligations) responsibilities (16%) (Statistics 2021). Two other factors associated with decreased consumption included higher education (Prowse et al. 2021)and using with parents (Bartel et al. 2020).

#### Age variation

Our review found 6 studies reporting on age variation of cannabis consumers during the pandemic. Key findings are reported here and in Table 7.

Compared to older adults, younger people were more likely to have increased their consumption since the start of the pandemic (Statistics 2021). Among respondents aged 15 to 29 who had previously consumed cannabis, 43% reported increasing their consumption during the pandemic (Statistics 2021). By comparison, 20% of respondents aged 50 to 64 and 22% of those aged 65 or older reported a consumption increase (Statistics 2021). Similarly, data from a cross-sectional study in Canada found significant increases in cannabis use among participants < 35 years compared to those ≥ 60 (Turner 2019). Findings from Turner et al. (2021) suggest that young people may be particularly impacted by behavior changes due to the early months of the pandemic.

Cross-sectional data collected between May to July 2020 from youth in grades 9 to 12 in Ontario and Secondary I–V in Quebec identified that the majority who consumed cannabis did not report increased cannabis use due to COVID-19 or using cannabis to cope with COVID-19 (Leatherdale et al. 2021). However, Leatherdale et al. (2021) found that although youth cannabis consumption in Canada increased between 2019 and 2020, the increase was not as high as expected had the pandemic not happened, supporting the claim that the pandemic had either slightly reduced the prevalence of consumption among youth in Canada or did not have a significant impact (Leatherdale et al. 2021).

#### Gender differences

Three studies reported key findings on differences in cannabis consumption between genders. These findings are reported here and in Table 8.

Dumas et al. found that, in April 2020, the percentage of cannabis-using adolescents aged 16–18 decreased for girls only (3% decrease, from 16 to 13%), and yet, the frequency of cannabis use (average number of cannabis using days) increased significantly for girls (Dumas et al. 2020), an increase which was not seen for boys.

The context of cannabis consumption also differed between genders. Dumas et al. found that increased cannabis use in the context of solidarity and face-to-face with peers was observed more in boys (58% and 31%, respectively) than girls (46% and 22%, respectively). Moreover, mixing cannabis with other substances was also more prevalent among boys (Dumas et al. 2020). On the other hand, more girls used substances with their parents (47%) and engaged with acquaintances through social posts (40%) in contrast to boys (30% and 24%, respectively).

The transition of in-person learning to online learning has significantly impacted the mental health of female students (25.4%) than male students (15.5%); however, male students reported a higher rates of cannabis use to cope with pandemic stress and schoolwork than their female counterparts (Prowse et al. 2021). Male students also reported using cannabis, alcohol, and vaping nicotine to cope with the negative impact of the pandemic including stress, mental health (Prowse et al. 2021), and increased hospitalization rate (Rotterman 2021).

#### Method of consumption

Our review found 2 studies that reported findings on methods of cannabis consumption during the pandemic which are explained here and in Table 9. While data is lacking on all methods of consumption during the pandemic, smoking was reported as the most commonly used method of consumption of cannabis for both males and females (Rotterman 2021; Bartel et al. 2020). Data collected by The National Cannabis Survey (NCS), which collected data from mid-November to December 31, 2020, reported that about 7 in 10 Canadians who reported using cannabis consumed dried flower or leaf (Rotterman 2021).

#### Health and safety impacts related to cannabis use during COVID-19

The indirect effects of the pandemic including the impacts of public health measures taken to control the spread of COVID-19 and the psychosocial impacts of the pandemic environment such as worry and fear have led to unintended health consequences during the pandemic (Canadian 2021). Two of the included studies reported findings on the health and safety impacts are detailed here and in Table10. The Canadian Institute of Health Information (CIHI) reported that more Canadians received substance-related hospital care during the COVID-19 pandemic than in the previous year. A substantial increase was observed in both emergency departments (ED) visits and hospitalizations for cannabis-related harms for the period of March to September 2020 compared with the same period in 2019. Specifically, there was an 8% increase in ED visits and a 5% increase in cannabis-related hospitalizations (Canadian 2021). Currie (2021) found adults who believed they would contract COVID-19 in the next year had increased their substance use (alcohol and/or cannabis) and thus reported more pandemic-related PTSD symptoms, than those who did not believe they would contract COVID-19 (Currie 2021). Data collected in June 2020 from 933 community-based adults in Alberta found high pandemic-related PTSD symptoms were associated with more than a twofold increase in the odds of increased substance use (alcohol and/or cannabis) in the past month among both women and men (Currie 2021).

## Discussion

In this scoping review, we identified 21 records that addressed the impact of the pandemic on patterns of use and cannabis-related health and safety in Canada. Our findings suggest that the impact of the pandemic on cannabis consumption in Canada is more complex than simplistic assumptions of increase or decrease. The association appears to be based on the characteristics of the consumers such as age, gender, type of user (occasional/frequent), reasons for use, and accentuated health impacts.

Our findings match those of other countries and portray a mixed picture with respect to cannabis use during the pandemic (Farhoudian et al. 2020; Turna 2021; Manthey et al. 2021; Vanderbruggen et al. 2020; van Laar et al. 2020; European 2021). An online survey of 21 European countries found that the pattern of change in cannabis consumption across Europe during the pandemic was not clear; however, on average, consumers reported an increase in use (Manthey et al. 2021). Similarly, a global survey found that approximately 42% of participating countries reported that cannabis consumption increased, whereas 26% and 22% reported a decrease and no change in cannabis use pattern, respectively (Farhoudian et al. 2020). Interestingly, a survey in Belgium found there were no significant changes in cannabis consumption noted in the period before and during the COVID-19 lockdown suggesting social isolation did not influence cannabis consumption (Vanderbruggen et al. 2020).

Similar to findings in Canada, cannabis consumption patterns in the Netherlands may have changed more among regular consumers during the pandemic (van Laar et al. 2020). In addition, data available from the European Monitoring Centre for Drugs and Drug Addiction (EMCDDA) suggests that some occasional users may have stopped using or reduced their cannabis use during the lockdown period, while those who had more frequent or intensive patterns of use may have increased their consumption (European 2021). These findings are similar to patterns seen in Canada (Imtiaz et al. 2021).

Motives that drove the increased consumption of cannabis during the pandemic appear to be similar around the globe. Top reasons reported for consuming cannabis more during the pandemic included boredom and loneliness (Vanderbruggen et al. 2020; van Laar et al. 2020). However, an interesting difference was noted when comparing healthcare practitioners in Italy, Brazil, and the USA to those in Canada where Canadian practitioners reported increasing their cannabis consumption during the pandemic to cope with stress and anxiety (Turna 2021).

Gender-specific considerations influencing cannabis consumption are important to consider for prevention and public education as the pandemic evolves. While the CCSA’s 2021 report “Differences in Cannabis Perceptions among Canadian Adolescent Boys and Girls’’ reported that boys and young men reportedly use cannabis more frequently and heavily than girls and young women (Goodman 2021), our scoping review found that in the context of COVID-19, cannabis use among female youth increased during the COVID-19 pandemic compared to male youth. Furthermore, there was a greater reduction in the expected escalation of cannabis use among males compared to females, suggesting cannabis consumption among males may be more socially driven than among females (Dumas et al. 2020; Leatherdale et al. 2021). As the effects of the pandemic extend beyond the disease itself, it has become imperative to understand the short and long-term consequences of the pandemic and understand the impacts for specific segments of the population, including variation between genders. Informing mitigation strategies with ​​a gender-specific understanding of patterns of use is important.

Smoking cannabis continued to be the most common method of consumption at the end of 2020. Evidence supports the concern that cannabis smoke can negatively affect heart and lung health (Canadian 2020). The extent to which cannabis smoke impacts respiratory and immune health in humans is not well-known and further studies are needed to confirm these relationships (Canadian 2020). This is concerning in the context of the pandemic as smoking cannabis has been linked to increased risk for COVID-19 complications and related death (Canadian 2020). In light of the ongoing pandemic, behaviors that might put an individual’s health at risk should be carefully considered. Furthermore, given smoking continues to be the most common method of consumption, it highlights a need for evidence-informed cannabis education, and in particular, with regards to Canada’s lower-risk cannabis use guidelines (Fischer et al. 2017).

Despite the effect of above factors on cannabis consumption during the pandemic, there are methodological issues that needs to be considered for interpretation and that are not explored in this study. The heterogeneity of findings could be due to difference in study designs and qualities. Due to the short timeframe between the pandemic and this study, follow-up length and baseline cannabis use could impact the consumption patterns that are captures in studies reviewed in this manuscript.

This scoping review identified several implications for future research and practice. Frequent cannabis consumers who are adolescents, young adults, mothers, and frontline workers require more attention for further research. The COVID-19 pandemic and measures to control it have caused a range of mental health issues leading to increased consumption to address those issues; however, this review did not find evidence of the benefits from consuming cannabis. Understanding the cycle of mental health and substance use is important to inform decisions and policies to facilitate greater availability of, and accessibility to, mental health and substance use services as the COVID-19 pandemic evolves and for future public health crises. In particular, further research is needed to understand the changes in cannabis consumption during the pandemic and how these changes have impacted health and safety that will be relevant post-pandemic. It would also be interesting to investigate the role of therapeutic cannabis use among regular cannabis users, during an unprecedented pandemic.

This scoping review includes a number of limitations. Many of the included studies were conducted relatively close to the beginning of the pandemic, therefore, routines may have not been established and the possible full impact of the pandemic not captured. Many of the studies reviewed used self-reported cannabis use and self-perceived changes during the pandemic. Subjectivity can be both a strength and a limitation, depending on different epistemological perspectives. In addition, the review is dependent on information on the review question being available and since the pandemic is ongoing there may be gaps in evidence. It is also possible that relevant sources of information may have been omitted. Finally, as is the case for all scoping reviews, no rating of quality or level of evidence is provided; therefore, practice recommendations cannot be graded.

## Conclusion

As we learn to live with COVID-19, its impact on and its relations with cannabis in Canada is an issue of importance. This scoping review synthesized rapidly growing knowledge on the relationship between the pandemic and cannabis consumption in Canada. This review suggested that the impact of the pandemic on cannabis consumption in Canada is more complex than simplistic assumptions of increase or decrease and it continues to be difficult to measure. The association depends on the characteristics of the consumers such as age, gender, reasons for increased and decreased use, and method of consumption. As the COVID-19 continues to evolve, high-quality evidence is needed to inform how public health-related policies and practices on cannabis can protect vulnerable groups such as young adults and frequent consumers, and future research should focus on better understanding considerations for mental health among cannabis consumers.

## Data Availability

The data can be made available upon request.

## Abbreviations

CCSA: Canadian Centre on Substance Use and Addiction
CCHS: Canadian Community Health Survey
CIHI: Canadian Institute for Health Information
CPSS: Canadian Perspectives Survey Series
MHCC: Mental Health Commission of Canada
MHRC: Mental Health Research Canada
NCS: National Cannabis Survey
EMCDDA: European Monitoring Centre for Drugs and Drug Addiction
